# Characterization of a Structural Intermediate of Flavivirus Membrane Fusion

**DOI:** 10.1371/journal.ppat.0030020

**Published:** 2007-02-16

**Authors:** Karin Stiasny, Christian Kössl, Jean Lepault, Félix A Rey, Franz X Heinz

**Affiliations:** 1 Institute of Virology, Medical University of Vienna, Vienna, Austria; 2 Laboratoire de Virologie Moléculaire et Structurale, UMR 2472/1157 CNRS/INRA, Gif-sur-Yvette, France; 3 Unité de Virologie Structurale, Institut Pasteur, Paris, France; The Rockefeller University, United States of America

## Abstract

Viral membrane fusion proceeds through a sequence of steps that are driven by triggered conformational changes of viral envelope glycoproteins, so-called fusion proteins. Although high-resolution structural snapshots of viral fusion proteins in their prefusion and postfusion conformations are available, it has been difficult to define intermediate structures of the fusion pathway because of their transient nature. Flaviviruses possess a class II viral fusion protein (E) mediating fusion at acidic pH that is converted from a dimer to a trimer with a hairpin-like structure during the fusion process. Here we show for tick-borne encephalitis virus that exposure of virions to alkaline instead of acidic pH traps the particles in an intermediate conformation in which the E dimers dissociate and interact with target membranes via the fusion peptide without proceeding to the merger of the membranes. Further treatment to low pH, however, leads to fusion, suggesting that these monomers correspond to an as-yet-elusive intermediate required to convert the prefusion dimer into the postfusion trimer. Thus, the use of nonphysiological conditions allows a dissection of the flavivirus fusion process and the identification of two separate steps, in which membrane insertion of multiple copies of E monomers precedes the formation of hairpin-like trimers. This sequence of events provides important new insights for understanding the dynamic process of viral membrane fusion.

## Author Summary

The fusion of cellular lipid membranes is an essential process in all forms of life. Such membranes are also part of a specific structural class of viruses—so-called enveloped viruses—that include influenza virus, HIV, severe acute respiratory syndrome coronavirus, Ebola virus, yellow fever virus, and many others. The fusion of the viral with a cellular membrane is a key step in the life cycle of these viruses and allows the delivery of their genetic information into cells. This entry step is controlled by specific proteins at the viral surface that are primed to undergo dramatic structural changes and thus drive membrane fusion. An interference with this process can be a powerful means for inhibiting virus replication and fusion inhibitors have recently become a valuable addition to the armamentarium of anti-HIV treatments. In the present study, we identified an intermediate of the fusion pathway of flaviviruses, which comprise mosquito- and tick-transmitted viruses such as yellow fever, dengue, West Nile, Japanese encephalitis, and tick-borne encephalitis viruses. This work has generated further insights into the mechanism of flavivirus membrane fusion and can thus provide new leads for the development of antiviral agents against these important human pathogens.

## Introduction

Membrane fusion processes are tightly regulated—spatially and temporally—by specific control proteins in both viral and cellular fusion systems [1–4]. Many enveloped viruses use only a single protein to mediate the fusion of their membrane with a cellular membrane during virus entry [3,4], which makes them a particularly interesting system for understanding the membrane fusion process in mechanistic terms. A common property of viral fusion proteins is their presence at the surface of mature virions in a metastable conformation that, when exposed to an appropriate trigger (receptor interactions, acidic pH, or a combination of both), undergoes structural rearrangements to drive the merger of the viral membrane with a membrane of the target cell (reviewed in [3]). In the course of these conformational changes, the fusion proteins expose a segment of the polypeptide chain (“fusion peptide” [FP]) that inserts into the cellular membrane to initiate the fusion process [4].

Distinct structural classes of viral fusion proteins have been identified, displaying radically different architectures and organizations on the virion [4–6]. Class I proteins, which form trimeric spikes, are found in orthomyxoviruses, paramyxoviruses, retroviruses, filoviruses, and coronaviruses. The class II proteins of flaviviruses and alphaviruses lie tangentially to the viral membrane and form an icosahedral oligomeric network at the virion surface [5,7,8]. A third category of fusion proteins with features of both class I and class II has been recently described for vesicular stomatitis and herpes simplex 1 viruses [9,10].

Despite the altogether different architecture of fusion protein classes, certain similarities in their overall postfusion conformation suggest that the corresponding fusion processes are mechanistically related [11,12]. A key feature in this context is the formation of a “hairpin”-like trimeric postfusion structure, bringing into contact the C-terminal membrane anchor with the target-membrane inserted FP [4,6,11,12]. The available crystal structures of the prefusion and postfusion conformations represent only snapshots at the start and at the end of a process that proceeds through a set of intermediate states [3,4,6,13]. A better understanding of the membrane fusion reaction demands the characterization of the postulated intermediates. Such intermediates have been identified for class I fusion proteins by the use of specific fusion inhibitors [14–20], by using modified recombinant forms of the fusion protein [21–24], or by the sequential application of different triggers (avian sarcoma and leukosis retroviruses) [19,20,25,26]. In the case of the class II alphavirus fusion protein E1, it was possible to stabilize an intermediate trimeric core by the addition of one of its domains in soluble form, which prevents the formation of the final postfusion hairpin-like structure and thus inhibits fusion [27].

**Figure 1:**
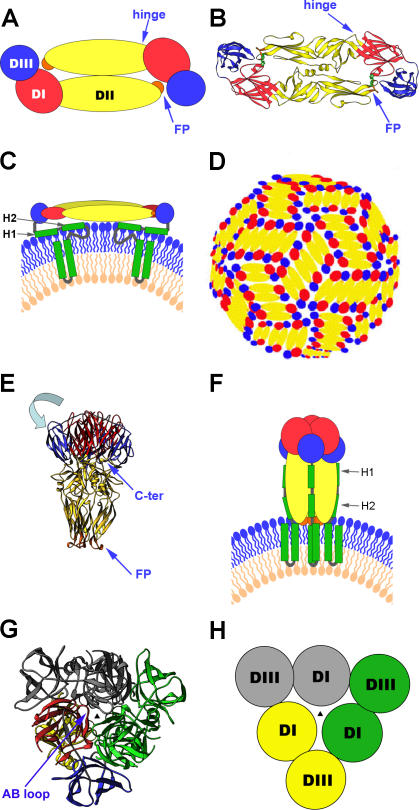
Summary of the Structural Organization and Different Conformations of the Flavivirus Envelope Protein E (A) Schematic top view of the organization of the sE protein dimer as present at the surface of mature virions, color-coded according to the three domains (DI, DII, and DIII). The FP is indicated in orange. (B) Crystal structure (top view) of the TBE virus sE dimer [31] represented as a ribbon diagram. (C) Schematic side view of the E dimer at the surface of mature virions, with the “stem” and TM C-terminal polypeptide segments (missing in the truncated sE form) indicated in green. The viral lipid bilayer is illustrated with lipids belonging to the outer and inner leaflets colored blue and pink, respectively. Cryoelectron microscopy 3D reconstructions have shown that the stem forms two α-helices (H1 and H2) lying on the viral membrane, followed by the two TM segments [37]. (D) Schematic diagram illustrating the icosahedral arrangement of E dimers at the surface of mature flavivirus particles—in a “herringbone” pattern—as determined for dengue and West Nile virus [29,30]. Ninety E dimers form a rigid glycoprotein cage enclosing the viral membrane. (E) Structure of the TBE virus sE in its trimeric postfusion conformation [33], represented as a ribbon diagram. Compared to the structure of E in the prefusion dimer, DIII is translocated (in a movement indicated by the light-blue arrow) to a lateral position, with its C terminus (labeled C-ter) projecting toward the FPs, thus generating a hairpin-like conformation. (F) Schematic representation illustrating the proposed organization of full-length E in its postfusion conformation. In this model, the α-helices of the stem interact with the body of the trimer, in the grooves between adjacent, parallel DIIs. The lipid bilayer as well as the stem and TM segments are drawn as in (C). (G) Top view of the sE trimer. For clarity, one of the subunits is colored according to domains, and the other two are given in a single color each (green and gray). The AB loop of DI (labeled in the figure [G]) rearranges upon dislocation of DIII, to make most of the DI–DI trimeric contacts. The relocated DIII acts as an external clamp, inserting into the grooves between DIs and providing additional intersubunit contacts. The vertical 3-fold axis at the center is indicated by a black triangle. (H) Schematic drawing to simplify the top view of the sE trimer, matching the color coding of (G) (except for the subunit in yellow, which corresponds to the one colored by domains in [G]), to highlight the trimer-stabilizing role of DIII in the hairpin-like conformation of the molecule.

In this work, we use tick-borne encephalitis (TBE) virus as a model to dissect the class II flavivirus membrane fusion pathway and the associated structural rearrangements of the envelope protein E. We found a monomeric conformation of E that can be postulated as a transient intermediate of the fusion pathway. TBE virus is a member of the flavivirus genus (family Flaviviridae) [28], which encompasses a number of other important human pathogens such as yellow fever, dengue, West Nile, and Japanese encephalitis viruses. Mature flavivirus particles are characterized by an external icosahedral glycoprotein cage formed by 90 dimers of E [29,30] (Figure 1A–1D) that completely cover the viral membrane and mediate both receptor-binding and membrane fusion. The crystal structures of the E ectodomain (termed “sE”) were determined for TBE and dengue viruses in both their prefusion and postfusion conformations [31–36]. In the prefusion form, as shown in Figure 1A and 1B, the three domains of sE are aligned along a rod-like molecule, with the C terminus and the FP lying at the two distal ends of the molecule [31,32,35,36]. In full-length E, the sE segment connects to the C-terminal transmembrane (TM) segments via an element of about 50 amino acids (called “stem”) that contains two α-helices, H1 and H2, which are peripherally attached to the viral membrane [37] (Figure 1C). The internal FP loop—located at the tip of domain (D)II—is buried at the dimer interface (Figure 1A and 1B). Dissociation of the E dimer into monomers, with concomitant exposure of the FP to allow its interaction with target membranes, occurs by breaking of the intradimeric and interdimeric contacts at the virion surface [38,39] (Figure 1D). Under physiological conditions, this process is triggered by the acidic pH in endosomes during receptor-mediated endocytosis and rapidly followed by E trimerization [40]. The structure of sE in the trimer (Figure 1E) shows that DIII is displaced from its original location and thus becomes positioned at the side of DI with its C terminus pointing toward the FP [33,34] (Figure 1E, 1G, and 1H). This movement results in the formation of a hairpin structure, suggesting that in the full-length postfusion form of E, the TM segments and the FP are juxtaposed in the fused membrane [33,34] (Figure 1F).

Here, we show that it is possible to trigger the conformational changes of E at the virus surface in discrete steps and, by trapping an intermediate, separate the initial target membrane insertion from downstream membrane fusion stages. Thus, E dimer dissociation with concomitant glycoprotein cage disassembly, allowing interactions of monomeric pre-hairpin intermediates with target membranes, can be induced without the subsequent E trimerization and completion of the fusion process. The monomeric conformation of E is obtained by exposing TBE virions to alkaline pH instead of the physiological acidic pH. Full fusion, however, can be induced by subsequent exposure to acidic pH, leading to hairpin formation and E trimerization, which drive the merger of the two lipid bilayers. The demonstration of a membrane-inserted prehairpin intermediate has important implications for the existing models of class II viral membrane fusion.

## Results

### Effect of Alkaline pH on the Virion Envelope Organization

**Figure 2:**
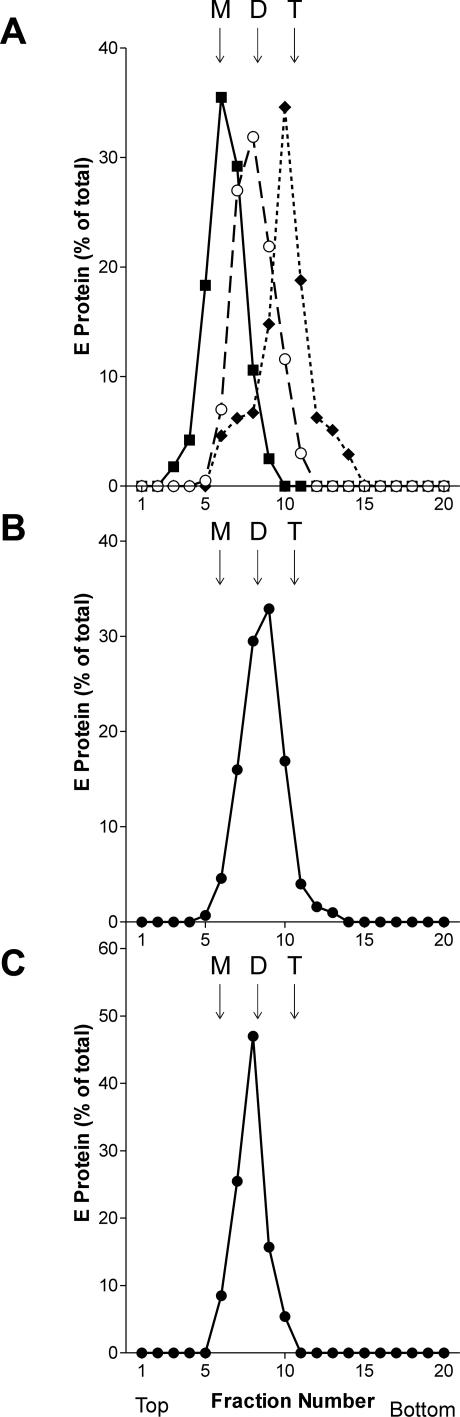
Sedimentation Analysis Demonstrating the Alkaline pH–Induced Dissociation of E and Its Reversibility (A) Virions were incubated for 10 min at pH 10.0 (boxes), pH 5.4 (diamonds), or pH 8.0 (circles), solubilized with Triton X-100, and analyzed at the respective pH by sedimentation in 7% to 20% sucrose gradients (w/w) containing 0.1% Triton X-100. (B) Material from the alkaline pH–induced E monomer peak in Figure 2A was back-neutralized to pH 8.0 and then centrifuged at pH 8.0 into 7% to 20% sucrose gradients (w/w) in buffer containing 0.1% Triton X-100. (C) Alkaline pH–treated virions were readjusted to pH 8.0, then solubilized with Triton X-100, and centrifuged at pH 8.0 in 7% to 20% sucrose gradients (w/w) in buffer containing 0.1% Triton X-100. The sedimentation direction is from left to right, and the positions of E monomer (M), dimer (D), and trimer (T) are indicated.

In addition to the physiological trigger, the fusogenic conformational change of a number of class I membrane fusion proteins can be induced by alternative treatments such as slightly denaturing conditions (exposure to elevated temperature or urea). This is the case for the influenza virus hemagglutinin [41] and the fusion proteins of paramyxoviruses [42,43] and retroviruses [44]. With TBE virus, in contrast, similar treatments had no functional effect and led to E protein denaturation only [45]. In a further attempt to identify alternative triggers and structural intermediates of flavivirus fusion, we investigated the effect of alkaline pH on the envelope organization and fusion-related processes of TBE virus. Purified virus samples in a pH 8.0 buffer were exposed to pH 10.0 and 5.4, respectively, solubilized with Triton X-100 and subjected to rate-zonal centrifugation, in order to compare the oligomeric state of E in these preparations. As expected, solubilization in Triton X-100 yielded E protein dimers at pH 8.0 and trimers at pH 5.4, whereas at pH 10.0, only E monomers were obtained (Figure 2A), indicating that alkaline pH causes a considerable weakening of the interaction forces of the E dimer. This dissociation was fully reversible upon back-neutralization, as revealed by experiments in which (i) the E monomer peak in Figure 2A was back-neutralized in the presence of Triton X-100 and (ii) virions were exposed to alkaline pH, back-neutralized, and solubilized with Triton X-100. In both cases, the analysis by sucrose density gradient centrifugation revealed a single peak at the position corresponding to the E dimer (Figure 2B and 2C).

**Figure 3:**
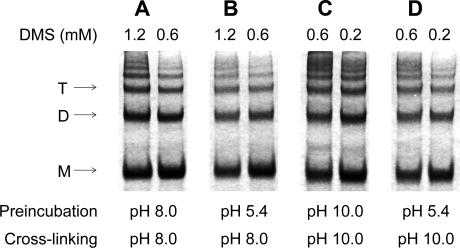
SDS-PAGE of TBE Virus Samples Cross-Linked with DMS at pH 8.0 and 10.0 without Pretreatment and after Pretreatment at pH 5.4 (A and B) SDS-PAGE of TBE virus samples cross-linked with DMS at pH 8.0 without pretreatment (A) and after pretreatment at pH 5.4 (B). (C and D) SDS-PAGE of TBE virus samples cross-linked with DMS at pH 10.0 without pretreatment (C) and after pretreatment at pH 5.4 (D). DMS concentrations are indicated on top of the individual lanes. Staining was performed with Coomassie blue. Positions of the E monomer (M), dimer (D), and trimer (T) are indicated.

We also attempted to confirm the oligomeric states of E at different pH values using chemical cross-linking without prior solubilization of the virus particles, as was used in the sedimentation analyses. However, a ladder of oligomeric bands was obtained in all cases (Figure 3), and their densitometric evaluation revealed continuously decreasing intensities from monomers to higher oligomers. This indicates that the density of the 180 E molecules in the viral membrane is apparently so high that the probability of the formation of intramolecular and intermolecular cross-links is similar, irrespective of the treatment at different pH values. Further conclusions as to the pH-induced changes in viral envelope organization could therefore not be based on this experimental approach.

**Figure 4:**
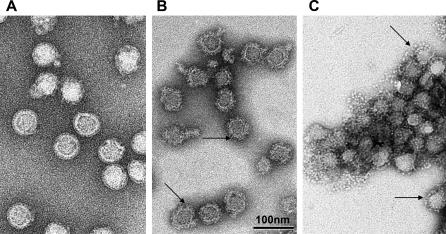
Electron Micrographs of TBE Virus at pH 8.0, 10.0, and 5.4 TBE virus was preincubated at pH 8.0 (A), 10.0 (B), and 5.4 (C), fixed with formalin, and negatively stained by phosphotungstic acid adjusted to pH 8.0 (samples A and B) or pH 6.0 (sample C). Arrows in (B) point to the rough surface generated by alkaline pH and in (C) to the bulky spikes generated by low pH treatment. All micrographs have been recorded at the same magnification. In (B) and (C), the virions lost their shell-like icosahedral envelope structure, at least at the particle surface, and as a consequence display irregular shapes that give the impression that the virus diameter is smaller than in (A). However, in all cases, the core diameter of the best-preserved virions has a similar value. In (C), the virions are aggregated, a characteristic of TBE virus maintained at low pH.

These changes, however, could be visualized using electron microscopy. In contrast to the smooth-surfaced native virions (Figure 4A), alkaline pH–treated particles exhibited a significantly rougher surface with radial projections. Taking into account the biochemical data obtained by density gradient centrifugation, these projections likely represent E monomers (Figure 4B). The control sample treated at low pH revealed heavily aggregated virus particles studded with bulky spikes corresponding to the trimeric “postfusion” structure of the E protein (Figure 4C).

### Target Membrane Binding of E Monomers at Alkaline pH

**Figure 5:**
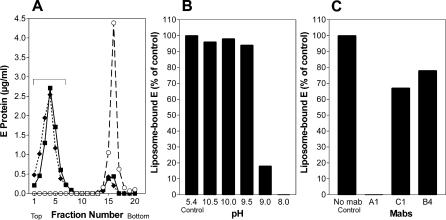
Analysis of the Interaction of TBE Virus with Target Membranes by Liposome Coflotation (A) Coflotation of virus with liposomes at alkaline pH. Virus was incubated for 10 min with liposomes at 37 °C at pH 10.0 (boxes) and as controls at pH 5.4 (diamonds) or pH 8.0 (circles), back-neutralized, and then subjected to centrifugation in sucrose step gradients. The gradients were fractionated, and the amount of E protein in each fraction was determined by a quantitative four-layer ELISA after denaturation of the samples with 0.4% SDS. The top fractions containing virus bound to liposomes are indicated by a bracket. (B) Percentage of E protein bound to liposomes at different pHs compared with the control at pH 5.4. (C) Percentage of E protein bound to liposomes at pH 10.0 after preincubation with different monoclonal antibodies compared with the control without monoclonal antibodies.

It was shown previously that the dissociation of the E dimer (induced by acidic pH under physiological conditions) is required for initiating membrane fusion because it exposes the internal FP and allows its interaction with the target membrane [39]. In order to assess whether the monomeric forms generated at alkaline pH were functionally equivalent to those generated at physiological pH with respect to this initial stage of the fusion process, we compared the binding of the virus to liposomes in a coflotation assay under both conditions. Aliquots of the virus preparation were mixed with liposomes at pH 8.0, adjusted to either pH 5.4 or 10.0, and subjected to centrifugation as described in Materials and Methods. Under both conditions, almost all of the E protein was recovered from the top of the gradient, indicating its association with the liposomes (Figure 5A). Further experiments were carried out to more precisely define the alkaline pH dependence of this association. As shown in Figure 5B, association was practically 100% between pH 10.5 and 9.5 but at pH 9.0 reached only about 20% of that observed at pH 5.4. Virtually no liposome association was found at pH 8.0, as expected.

From the experiments described, it was not yet clear whether the interaction with liposomes at alkaline pH was indeed mediated through the internal FP loop, as is the case at acidic pH [38,39]. We therefore assessed whether coflotation could be inhibited by a monoclonal antibody (designated A1), which is specific for the FP loop [38]. Two antibodies reactive with DI (C1) and DIII (B4) were used as controls. The result was similar to that obtained previously at acidic pH [39]; i.e., only the binding of monoclonal antibody A1 resulted in complete blocking of membrane binding (Figure 5C), suggesting that the FP loop is responsible for the interactions with liposomes at both pH values. In earlier studies [39,46], it was shown that the interaction of the FP loop with target membranes at acidic pH could facilitate the formation of trimers with a soluble C-terminally truncated E (sE) that, in the absence of liposomes, only dissociates into its monomeric constituents and does not trimerize. In the case of alkaline pH, however, no evidence for E trimerization induced by virus attachment to liposomes was obtained, and sedimentation analysis in the presence of Triton X-100 revealed only E monomers (unpublished data).

### Can Fusion Be Induced at Alkaline pH?

**Figure 6:**
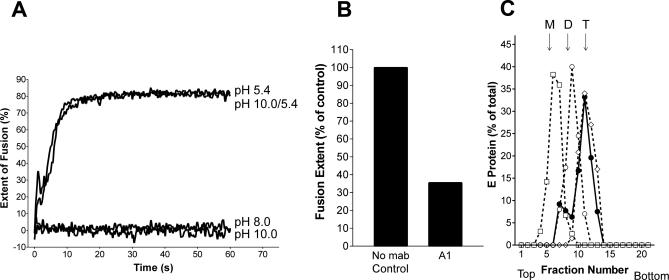
Analysis of Membrane Fusion, E Dissociation, and Trimerization at Different pH Values (A) Fusion of pyrene-labeled TBE virus with liposomes was carried out at 37 °C at the following conditions: (i) virus and liposomes were mixed an adjusted to pH 5.4, (ii) virus and liposomes were mixed and kept at pH 8.0, (iii) virus and liposomes were mixed and adjusted to pH 10.0, and (iv) virus and liposomes were preincubated at pH 10.0 for 10 min before adjustment to pH 5.4. The corresponding curves are labeled pH 5.4, pH 8.0, pH 10.0, and pH 10.0/5.4, respectively. (B) Extent of low pH–induced fusion of pyrene-labeled TBE virus pretreated at pH 10.0 in the absence (control) and the presence of the FP-specific monoclonal antibody A1. The figure shows the values obtained 1 min after acidification. (C) Sedimentation analysis demonstrating the low pH–induced trimer formation of virions preincubated at pH 10.0. Virions and liposomes were preincubated at pH 10.0 for 10 min, adjusted to pH 5.4 (filled circles), solubilized, and subjected to sucrose density centrifugation as described for Figure 2. As controls (dotted lines), virions were incubated for 10 min at pH 10.0 (boxes), pH 5.4 (diamonds), or pH 8.0 (open circles), solubilized, and analyzed as described above. The sedimentation direction is from left to right, and the positions of E monomer (M), dimer (D), and trimer (T) are indicated.

Because dissociation of the E dimer into monomers and their interaction with the target membrane via the FP loop are the first steps in the proposed flavivirus membrane fusion cascade [39,47], we investigated whether alkaline pH can substitute for acidic pH to cause membrane fusion. The experiments were carried out at pH 5.4, 8.0, and 10.0 using an in vitro liposome fusion assay with pyrene phospholipid–labeled virus and unilamellar liposomes (see Materials and Methods). In contrast to the efficient fusion induced at pH 5.4, the results obtained at pH 10.0 were negative and comparable to the control at pH 8.0 (Figure 6A), indicating that the dissociation of the dimeric E protein and its interaction with the target membrane alone were insufficient to drive the fusion process to completion.

We further analyzed whether the E protein in particles bound to liposomes at alkaline pH was still competent for fusion at acidic pH. For that purpose, pyrene-labeled virions were first allowed to interact with liposomes at pH 10.0 for 10 min at 37 °C, and then the mixture was acidified to pH 5.4 and fusion was monitored as described. As can be seen from Figure 6A, the extent and rate of fusion were the same as observed in the control. The addition of the FP-specific monoclonal antibody A1 strongly impaired the efficiency of fusion under these conditions (Figure 6B), consistent with the dissociation of the E dimer and concomitant exposure of the FP at alkaline pH.

We also determined the oligomeric state of E after low pH–induced fusion of alkaline pH–pretreated virus using Triton X-100 solubilization and rate zonal centrifugation (Figure 6C). As expected from the fusion results, the E proteins were almost quantitatively converted into trimers, similar to those obtained without alkaline pH pretreatment. From these experiments, it can be concluded that the alkaline pH–mediated attachment of E monomers to target membranes leaves the proteins in a state that is still competent for fusion upon acidification. In addition to its fusion activity, the other entry functions of E in virions are apparently preserved or can be completely restored after alkaline pH treatment. Virus preparations exposed to pH 10.0 for 30 min at 37 °C and back-neutralized to pH 7.6 did not exhibit any reduction in infectivity titers in BHK-21 cells compared with untreated controls (5 × 10^7^ focus-forming units/ml in both cases).

### Electron Microscopy of Virus–Liposome Interactions

**Figure 7:**
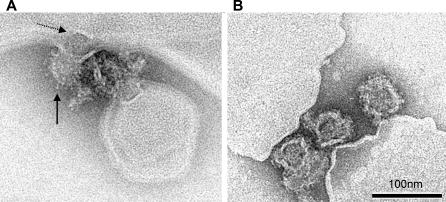
Electron Micrographs of TBE Virus Interacting with Liposomes at pH 5.4 and 10.0 (A) Electron micrographs of a virus particle in the process of low pH–induced fusion with a liposome. Solid arrow points to low pH–induced projections at the virion surface; dotted arrow points to an electrodense structure presumed to be the nucleocapsid in the process of release. (B) Virus particles attached to liposomal membranes at alkaline pH. Negative stain by phosphotungstic acid adjusted to pH 8.0.

For the visual representation and distinction of low pH–induced membrane fusion and alkaline pH–induced membrane attachment, mixtures of virions and liposomes were treated at pH 5.4 and 10.0, respectively, fixed with formalin and subjected to electron microscopy as described in Materials and Methods. Figure 7A shows a snapshot of a virion in the process of direct acidic pH–induced membrane fusion. The viral and liposomal membranes are contiguous, and an electron-dense structure (possibly the nucleocapsid) appears to be in the process of being released into the interior of the liposome. These bulky spikes observed throughout the surface of the fusing virion suggest that those E proteins in the particle that cannot contact the target membrane form postfusion trimers and insert their fusion loops into the viral membrane, similar to what has been described for influenza virus [48]. With the alkaline pH–treated samples, on the other hand, only particles attached to, not fused with, liposomal membranes could be detected (Figure 7B). These images are compatible with an attachment through the distal ends of monomeric E projections.

## Discussion

The current models of viral membrane fusion comprise several successive steps and the formation of intermediate fusion protein structures that play different roles in the initiation and completion of the fusion process [3,4]. For class I viral fusion, such intermediates have been described both at the structural level in relation to the fusion protein (“prehairpin intermediates”) and at the mechanistic level in relation to the merger of the membranes (“hemifusion”) [6,13]. Their identification was certainly facilitated by the fact that the conformational transitions and concomitantly the class I–mediated fusion process are quite slow (half-life of several minutes) and therefore provides a long window of opportunities for the interaction of inhibitors with structural fusion intermediates [49,50]. The fusion machinery of flaviviruses, in contrast, is extremely fast (half-life of about 2 s) [51], and the trapping of intermediates or the inhibition of fusion by similar principles has not yet been described. As shown in our study, however, it is possible to separate the flavivirus fusion process into two discrete stages by the application of alkaline pH, which triggers only the formation of a prehairpin intermediate and its interaction with target membranes but not hairpin formation and membrane fusion.

The smooth icosahedral glycoprotein cage of mature virions is sensitive to the environmental pH being stable in a limited pH range and opens up when exposed to either an alkaline or acidic milieu. Such “opening up” of the cage appears to be caused by simultaneous dissociation of the 90 E dimers that at neutral pH are interlocked in a “herringbone” pattern at the virus surface [29,30] (Figure 1D). As suggested by solubilization and sedimentation analyses and the rough contour of alkaline pH–treated virions in electron micrographs, alkaline pH treatment results in E monomers protruding from the surface and thus exposing their FP loops at the distal tip. Such a pattern is likely to allow an efficient multivalent interaction of the FP with the target membrane. Indeed, the high avidity of the interaction resulting from multiple E protomers is reflected in the capacity of the particles to coflotate with liposomes, whereas the same test using isolated sE dimers exposed to alkaline pH results in no detectable binding to membranes (unpublished data). This difference could be due to the lack of the stem-anchor region in sE, but inhibition studies with an FP-specific monoclonal antibody support the interpretation that the observed virion–liposome interactions at alkaline pH are indeed mediated by the FP loop, analogous to the interactions observed when E dimers dissociate under physiological conditions at acidic pH [39].

What is the likely conformation of the E monomers at the virus surface upon dimer dissociation? The “hinge” region between DI and DII (Figure 1A and 1B) has been reported to allow different angles between these two domains in different crystal structures of the dengue virus E protein, depending on the crystal packing [32,35,36]. A slight bend between DI and DII is apparently needed in the sE dimer for the FP to fit in a pocket between DI and DIII in the partner subunit (Figure 1A and 1B). Structural studies on the homologous protein from Semliki Forest virus, which has been crystallized as a monomer, suggest that in the “relaxed” form, i.e., in the absence of oligomeric interactions, the molecule forms a straight rod [5,52]. It is therefore likely that the sE segment also forms a straight rod in the monomeric form and that the flexibility at the connection between DIII and H1 (Figure 1C) is sufficient for E to project from the virion surface.

There is experimental evidence that intermediates of the E dimer–trimer transition interact with target membranes [39], but it was not clear whether trimerization can occur before DIII relocation and hairpin formation. Inspection of the postfusion trimer shows that the most important trimer contacts are provided by (i) the AB loop in DI in a conformation that is only generated after displacement of DIII (Figure 1G) and (ii) interactions of the external surface of DI with relocated DIII, which functions as an external clamp for stabilizing the trimer (Figure 1E–1H). These structural considerations together with our experimental data thus favor the conclusion that the initial target membrane binding is mediated by multiple copies of monomeric E intermediates in their prehairpin conformation and that DIII relocation and trimerization are concomitant. Although alkaline pH treatment leads to E dimer dissociation and thus generates monomeric intermediates, it apparently does not disrupt the intramolecular DI–DIII interactions required for DIII relocation. Based on the presence of two strictly conserved histidine residues at the DI–DIII contact site (His146 in DI and His323 in DIII), we had postulated previously that protonation of these side chains by acidic pH may be important for the destabilization of the DI–DIII interactions in the protomer [33]. The observed inhibition of dengue virus fusion by soluble DIII present during the low pH treatment [27] can be explained by the fact that exogenous DIII is unconstrained and can bind and stabilize an intermediate form of E before the endogenous DIII can reach its correct location to form a hairpin. In electron micrographs taken at acidic pH, the virus particles display thick knob-like radial projections consistent with the structure of E trimers in their postfusion conformation. This suggests that the E protomers not interacting with the target membrane trimerize and insert their FPs into the viral membrane through the relocation of DIII and concomitant placement of the stem into lateral grooves (as diagrammed in Figure 1F).

The formation of a monomeric prehairpin intermediate suggests a new possibility: because only a subset of the E protomers can reach and interact with the target membrane, it cannot be ruled out that their subsequent trimerization may lead to “mixed trimers,” with individual subunits inserted into the target and others into the viral membrane. Such mixed trimers would have an even stronger destabilizing effect on the two membranes, greatly favoring the generation of a hemifusion stalk [53] and the subsequent completion of the membrane fusion reaction.

In the case of flaviviruses, the involvement of a monomeric intermediate was anticipated because membrane fusion requires a rearrangement of E dimers into trimers [53]. Similarly, trimerization and hairpin formation of the related class II fusion protein E1 of alphaviruses also requires the prior dissociation into a monomeric form, in this case from its heterodimeric partner E2 in mature virions (reviewed in [54]). Experiments in the presence of Zn^2+^ ions [55] and with FP loop mutants [56] have indeed provided evidence that the interaction of E1 with target membranes—as we have shown for TBE virus E—can occur in the absence of trimer formation, suggesting similar pathways of membrane fusion. Even in the case of class I fusion proteins, in which the oligomeric state is trimeric in both conformations, there are important differences in the trimer interfaces of their prefusion and postfusion forms [57,58], and a putative transient dissociation of the prefusion trimer cannot be ruled out. Further data are obviously needed to test the universality of our observations on flavivirus fusion.

## Materials and Methods

### Virus growth and purification.

The TBE virus prototype strain Neudoerfl was grown in primary chicken embryo cells, harvested 48 h after infection, and purified by two cycles of sucrose density gradient centrifugation [59]. For membrane fusion assays, virions were metabolically labeled with 1-pyrenehexadecanoic acid as described previously [51].

### Liposomes.

For the preparation of liposomes, phosphatidylcholine (Avanti Polar Lipids, http://www.avantilipids.com), phosphatidylethanolamine (Avanti Polar Lipids), and cholesterol (Sigma Chemical Co., http://www.sigmaaldrich.com) were mixed at a molar ratio of 1:1:2 from stock solutions in chloroform [60]. The mixture was dried to a thin film in high vacuum for at least 1.5 h. The lipid film was hydrated in liposome buffer (10 mM triethanolamine, 140 mM NaCl [pH 8.0]) and subjected to five cycles of freeze-thawing, followed by 21 cycles of extrusion through two polycarbonate membranes with a pore size of 200 nm using a Liposofast syringe-type extruder (Avestin, http://www.avestin.com).

### pH treatments.

Purified TBE virions in TAN buffer (50 mM triethanolamine TEA, 100 mM NaCl [pH 8.0]) were (i) adjusted to different alkaline pH values (pH 9.0, 9.5, 10.0, or 10.5) with 200 mM 3-[cyclohexylamino]-1-propanesulfonic acid (CAPS) buffer (containing 100 mM NaCl) that had been pretitrated to yield the desired final pH, (ii) acidified with 300 mM morpholinoethansulfonic acid (MES) to reach pH 5.4, or (iii) kept at pH 8.0 in TAN buffer. The samples were then incubated for 10 min at 37 °C at the respective pH. For the analysis of effects of alkaline pH treatment and back-neutralization, the samples were readjusted to pH 8.0 using 50 mM MES. Low pH–treated samples were back-neutralized with 150 mM triethanolamine. For cross-linking at pH 10.0, the CAPS buffer was replaced by 0.2 M TEA and adjusted with 1N NaOH to the final pH as required.

### Sedimentation analysis.

The oligomeric state of E after the exposure of virions to different pHs was determined by sedimentation analysis in sucrose gradients as described previously [40,60]. Purified virions (3 to 5 μg) in TAN buffer were incubated for 10 min at 37 °C at pH 10.0, 8.0, or 5.4 and solubilized with 1% Triton-X 100. For some experiments, pH 10.0–pretreated samples were back-neutralized to pH 8.0 or acidified to pH 5.4 before solubilization. The samples were then applied to 7% to 20% sucrose gradients (w/w) in the respective buffer containing 0.1% Triton-X 100 and were centrifuged for 20 h in an SW 40 rotor (Beckman Coulter, http://www.beckmancoulter.com) at 38,000 rpm and 15 °C. Fractions were collected by upward displacement, and the amount of E in each fraction was determined by four-layer ELISA after denaturation with 0.4% SDS at 65 °C [61].

### Chemical cross-linking analysis.

Purified virus preparations at a concentration of 9 μg/ml were cross-linked with dimethyl suberimidate (DMS; Pierce Biotechnology, http://www.piercenet.com) at pH 8.0 and pH 10.0 with or without pH 5.4 pretreatment for 10 min at 37 °C. DMS was added to final concentrations of 1.2 mM and 0.6 mM at pH 8.0 and (because of the higher efficiency of cross-linking) 0.6 mM and 0.2 mM at pH 10.0. After incubation for 30 min at room temperature, the reaction was stopped by the addition of ethanolamine to the same final molar concentrations as DMS. The cross-linked virions were then concentrated by pelleting in a Ti90 rotor (Beckman) at 50,000 rpm for 1 h at 4 °C. The pellets were resuspended, solubilized in SDS-containing sample buffer and subjected to SDS-PAGE using 5% polyacrylamide gels according to Maizel [62]. Staining was performed with Coomassie blue R-250.

### Coflotation of virions with liposomes.

Virions were mixed with liposomes at a ratio of 1 μg of virus to 100 nmol of lipid. The virus–liposome mixture was incubated for 10 min at a virus concentration of 45 μg/ml and 37 °C at alkaline pH (pH 9.0, 9.5, 10.0, and 10.5) and adjusted to 20% (w/w) sucrose in CAPS buffer (50 mM CAPS, 100 mM NaCl) at the corresponding pH in a final volume of 2 ml as described previously [39]. For controls, the virions were incubated in the presence of liposomes at pH 5.4 (positive control) or pH 8.0 (negative control). The 2-ml virus–liposome mixture was then applied to a 50% cushion and overlaid with 1 ml of 5% (w/w) sucrose. Centrifugation was carried out for 1.5 h at 50,000 rpm at 4 °C in a Beckman SW 55 rotor, and fractions were collected by upward displacement. The amount of E protein in each fraction was determined by a quantitative four-layer ELISA as described above.

For the inhibition of coflotation by monoclonal antibodies, virions (final protein concentration 37.5 μg/ml) were incubated with purified E protein–specific monoclonal antibodies (final protein concentration 225 μg/ml) at 25 °C for 30 min at pH 8.0 or pH 10.0 before the addition of liposomes.

### Electron microscopy.

Purified virions (80 μg/ml) at pH 10.0, 5.4, or 8.0—in the presence or absence of liposomes—were fixed with formalin (final dilution 1:2,000) for 24 h at 37 °C. A drop of the virus or virus–liposome suspensions was deposited on an air-glow discharged carbon-coated grid. The drop was blotted with filter paper, and the grid was washed with a 2% phosphotungstic acid solution adjusted to pH 6.0 (sample acidic pH) or 8.0 (samples at pH 8.0 and 10.0). The grids were observed in a CM12 electron microscope (Philips, http://www.philips.nl) operated at 80 kV, and the micrographs were recorded on Kodak SO163 image plates developed for 5 min in D19.

### Fusion assay.

Fusion of pyrene-labeled virions with liposomes was measured by monitoring the decrease in pyrene excimer fluorescence caused by the dilution of pyrene-labeled phospholipids in the viral membrane into the unlabeled liposome membrane [51,60]. Fluorescence was recorded continuously for 60 s at 480 nm using a Perkin Elmer (http://www.perkinelmer.com) LS 50B Fluorescence Spectrophotometer at an excitation wavelength of 343 nm. Pyrene-labeled virions (0.5 to 1 μM) were mixed with 0.3 mM liposomes at pH 8.0 in a continuously stirred fluorimeter cuvette at 37 °C, and different pH values were adjusted by the addition of 150 mM CAPS (alkaline pH) and 300 or 600 mM MES (acidic pH). The initial excimer fluorescence after mixing was defined as 0% fusion. To determine the residual excimer fluorescence at infinite dilution of the probe (defined as 100% fusion for calculating the fusion extents), the detergent *n*-octa(ethylene glycol)-*n*-dodecyl monoether was added to a final concentration of 10 mM to disperse the viral and liposomal membranes.

For the inhibition of fusion by monoclonal antibody pyrene-labeled virions (final protein concentration 37.5 μg/ml) were incubated with purified E protein–specific monoclonal antibodies (final protein concentration 225 μg/ml) at 25 °C for 30 min at pH 10.0 before the addition of liposomes.

## Supporting Information

### Accession Numbers

The GenBank (http://www.ncbi.nlm.nih.gov/Genbank) accession number for the flavivirus TBE virus strain Neudoerfl is TEU27495.

## Abbreviations

D: domain
FP: fusion peptide
TBE: tick-borne encephalitis
TM: transmembrane
